# Effects of Shared and Nonshared Schizophrenia and Bipolar Disorder Alleles on Cognition and Educational Attainment in the UK Biobank

**DOI:** 10.1016/j.bpsgos.2025.100601

**Published:** 2025-08-22

**Authors:** Alexander L. Richards, Eilidh Fenner, Nicholas E. Clifton, Darren Cameron, Claire E. Tume, Nicholas J. Bray, Sophie E. Legge, James T.R. Walters, Peter A. Holmans, Michael C. O’Donovan, Michael J. Owen

**Affiliations:** a Centre for Neuropsychiatric Genetics and Genomics, Division of Psychological Medicine and Clinical Neurosciences, School of Medicine, Cardiff University, Cardiff, United Kingdom; b Department of Clinical & Biomedical Sciences, Faculty of Health and Life Sciences, University of Exeter, Exeter, United Kingdom; c Neuroscience & Mental Health Innovation Institute, Cardiff University, Cardiff, United Kingdom

**Keywords:** Bipolar disorder, Cognition, Education, Enrichment, Genomics, Schizophrenia

## Abstract

**Background:**

Cognitive impairment is typically more severe in schizophrenia (SZ) than bipolar disorder (BD). We explored the underlying genetics and biology of this difference and its relationship to educational attainment (EA) using genomic structural equation modeling.

**Methods:**

Shared and differentiating fractions of liability for SZ and BD were derived and tested for their association with general intelligence (*n* = 93,541), fluid intelligence (*n* = 160,465), and EA (*n* = 354,609) in the UK Biobank. Liabilities were tested for enrichment in genes with high expression specificity (HES) for developmental stages, cell types, and functional categories.

**Results:**

Shared liability was associated with poorer cognition but higher EA. The SZ differentiating fraction (SZ_diff_) was associated with poorer cognition and lower EA. When we adjusted for cognition, the effects of SZ_diff_ on EA were attenuated but still significant. The differentiating fraction was enriched for HES genes for young adulthood (20–30 years), mid-adulthood (30–60 years), and the dentate gyrus.

**Conclusions:**

Shared liability for SZ and BD is enriched for alleles that confer risk for poorer cognitive function in the general population but is associated with noncognitive traits that enhance EA. In contrast, SZ_diff_ is enriched for alleles that confer risk for poorer EA through both cognitive and noncognitive mechanisms, which has implications for interventions. The enrichment of the differentiating fraction for HES genes in early and mid-adulthood and in the dentate gyrus highlights developmental stages and cell types important for future research.

## Plain Language Summary

We identified genetic risk that is either shared by schizophrenia and bipolar disorder or differentiates them. We found that shared risk is associated with poorer cognitive function in the general population but with noncognitive traits that enhance educational attainment. Differentiating risk for schizophrenia was associated with poorer cognition, lower educational attainment via both cognitive and noncognitive mechanisms, and specific developmental stages and cell types.

Schizophrenia (SZ) and bipolar disorder (BD) are highly heritable polygenic conditions (1). Although they are distinct entities in major diagnostic systems (2,3), their clinical features nevertheless overlap (4), as do their genetic liabilities, with a genetic correlation (*r*_g_) of around 0.7 (5). This is consistent with SZ and BD occupying different, but overlapping, positions on several dimensions of psychopathology rather than being fully independent categories of disorder (6). This hypothesis is further supported by findings that risk alleles that influence major dimensions of symptomatology (e.g., psychosis, depression, mania) are partially distinct and influence those dimensions across diagnoses (7, 8, 9, 10).

Cognitive impairment is typically more severe in SZ than in BD (11, 12, 13), involves many aspects of cognitive function (14) including general intelligence (*g*), and is qualitatively similar (15, 16, 17). While consistent with a dimensional view, this suggests that there may be pathogenic processes manifested by cognitive impairment that are more prominent in individuals diagnosed with SZ. Cognitive impairment is strongly associated with functional outcomes (14) and therefore is of considerable importance. Understanding its etiology in SZ and BD may point to potential interventions that could improve outcomes (18) or even prevent illness onset if low cognitive ability were established as a causal risk factor.

It has been postulated that the cognitive impairment seen in SZ reflects an underlying perturbation of neurodevelopment that is more prominent in SZ than in BD (19,20). This implies that alleles that preferentially increase liability to SZ over BD are enriched for variants associated with poorer cognition and in genes whose expression characterizes early brain development.

Genomic structural equation modeling (gSEM) (21) is an adaptation of SEM that allows genetic liability that is shared between 2 or more genetically correlated traits to be extracted from input genome-wide association study (GWAS) summary statistics of the individual traits. It then allows the fraction of liability to each individual trait that is not included in the shared component to be isolated from the input GWAS, which is the component of liability that is specific to each trait. Here, to test our predictions, we applied gSEM to GWASs of SZ and BD to isolate the fraction of common variant liability that is shared by these two disorders as well as that fraction that differentiates between them. We used genetic correlation and polygenic risk score (PRS) methods to examine the relationships between these fractions and cognition in a population sample without SZ or BD. We also sought to identify functional gene sets, cell populations, and developmental time points that are enriched for the differentiating fraction of liability.

Finally, we examined the relationships between genetic liability to SZ and BD, cognitive ability, and educational attainment (EA). Our motivation here was 2-fold. First, EA is often used as a proxy measure of cognitive ability in genomic studies. Second, some (22, 23, 24, 25) though not all (26, 27, 28) studies have reported the surprising finding that genetic liability to SZ shows a small positive association with genetic liability for educational outcomes despite the robust evidence for both lower cognitive ability and poorer educational outcomes in SZ (29).

## Methods and Materials

See Supplemental Methods for additional information.

### Genomic SEM

GWAS summary statistics came from studies of SZ and BD conducted by the Psychiatric Genomics Consortium (PGC) (30,31) (Table S1; all input samples of European ancestry). Single nucleotide polymorphisms (SNPs) present in both studies [minor allele frequency >1% in HapMap 3 (32), imputation score >0.7, MHC region excluded] were retained (*N* = 7,334,582). We ran gSEM in R (version 4.0.3; The R Foundation) using the GenomicSEM package (21) to apply a common factor model to the GWAS summary statistics. For each SNP, the loading on the common factor was extracted to produce a statistic corresponding to the effect shared between disorders. We then applied a model extracting the loading of each SNP on the residual variance from each input GWAS that was not explained by the common factor so that the residual effect sizes for each SNP indexes its influence on the probability of having one phenotype over the other (see lavaan models in Supplemental Methods).

For the SZ differentiating fraction (SZ_diff_), alleles with effects signed above 0 increase the probability of SZ over BD, while those below 0 indicate the converse. For the BD differentiating fraction (BD_diff_), alleles with effects signed above 0 increase the probability of BD over SZ, while those below 0 indicate the reverse. As there are only 2 phenotypes in the model, SZ_diff_ and BD_diff_ are perfectly negatively correlated. We use the terms SZ_diff_ and BD_diff_ when we are presenting results where a direction of effect is meaningful, for example when testing against *g* or EA, so that it can be understood whether the alleles that favor development of one of the disorders are associated with higher or lower *g* or EA. For gene set enrichment, the direction of effect does not affect the results, and therefore we use the term differentiating (diff) to refer to the results for the differentiating factors.

SNP-based heritability (*h*^2^_SNP_) and genetic correlations were calculated using linkage disequilibrium (LD) score regression (33,34).

### Cognitive and Education Datasets

We tested for genetic correlations between the input GWAS and gSEM fractions and summary statistics (35,36) for *g* and EA. We also used a PRS approach (37) to test for associations between gSEM fractions of liability and measures of cognition and EA in the UK Biobank (UKBB), a UK prospective volunteer study of approximately 500,000 participants ages 40 to 69 years at the time of recruitment (http://www.ukbiobank.ac.uk). The North-West Multi-Centre Ethics Committee granted ethical approval to the UKBB, and all participants provided written informed consent. This study was conducted under UKBB Project No. 13310.

### Genotyping and Phenotyping in the UKBB

See Supplemental Methods for full variant and individual exclusion criteria.

Individuals with a diagnosis of BD, SZ, or a psychotic disorder were excluded (38). *g* was derived as a measure of general intelligence from the standardized first principal component of 4 cognitive measures (numeric memory, reaction time, pairs matching, and Trail Making Test B) (Table S2). We used the measures of fluid intelligence (FI) and the highest EA provided in the Biobank data. The EA variable was transformed into an ordinal measure (26).

### PRS Analyses

PRSs were derived as described (37) on clumped SNPs without thresholding on *p* values. We tested standardized PRSs for association with *g* (*n* = 93,541) (Table S3) and FI (*n* = 160,465) using linear regression. PRSs were also tested for associations with EA using ordinal regression (*n* = 354,609).

### Developmental Stage Enrichment Analyses

Transcriptomic data from the human dorsolateral prefrontal cortex and hippocampus, aged between 12 postconception weeks and 84 years, were obtained from BrainSeq phase II (39). Samples were divided into 10 developmental stages, and for each gene, a *t* statistic was calculated as a measure of expression specificity in one stage relative to all other ages (Table S4) (40,41). The top 10% genes, ranked by their specificity *t* statistics, were selected to define high expression specificity (HES) gene sets for each stage, which were then tested for enrichment of *h*^2^_SNP_ in the gSEM fractions (as well as source GWAS data for comparison) using stratified LD score regression version 1.2 (33,42). The 1-sided coefficient *z* score *p* value, accounting for 53 baseline genomic annotations, was used to indicate significance.

### Cellular Enrichment Analyses

Cellular gene expression specificity scores were obtained for cell populations from human fetal brain (43), human prefrontal cortex spanning gestation to adulthood (44,45), adult human frontal cortex and hippocampus (46), entire adult human brain (47,48), and mouse brain (49) (Table S5). Specificity scores were calculated in the cited studies by dividing each gene’s expression in a given cell type by the sum of that gene’s expression across all cell types. As above, the top 10% of HES genes for each cell type were tested for heritability enrichment using stratified LD score regression version 1.2 (31,48).

### Gene Ontology Enrichment Analyses

We tested for enrichment of gSEM and GWAS associations in gene ontology (GO) term gene sets using MAGMA (version 1.10) (50). GO terms were downloaded from the Gene Ontology Consortium (51,52). One-sided competitive *p* values for each GO term were extracted as the primary test statistics.

## Results

### Heritability and Genetic Correlations

**Figure 1:**
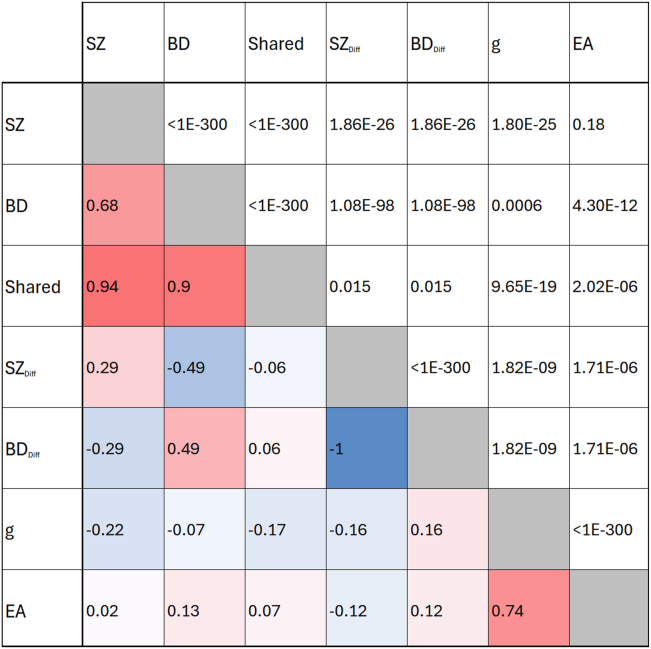
Heatmap showing genetic correlation for SZ (31) and BD (30) GWASs, genomic structural equation modeling shared and SZ_d__iff_ fraction (derived in the current study) and published *g* (35) and EA (36) GWASs from general population samples. Correlations were calculated using linkage disequilibrium score regression. Genetic correlation (*r*_g_) values are below the diagonal. Genetic correlation *p* values are given above the diagonal. BD, bipolar disorder; Diff, differentiating fraction; EA, educational attainment; *g*, general intelligence; GWAS, genome-wide association study; SZ, schizophrenia.

Estimated *h*^2^_SNP_ and genetic correlations are shown in Table 1 and Figure 1. As expected from the known strong genetic correlation between SZ and BD, most heritability from gSEM-derived components was assigned to the shared fraction.

**Table 1:** SNP Heritability of Schizophrenia (31) and Bipolar Disorder (30) GWASs and gSEM Fractions

GWAS	SNP Heritability	SE
Schizophrenia	0.35	0.01
Bipolar Disorder	0.28	0.01
Shared	0.26	0.01
Differentiating	0.14	0.01

SNP heritability is reported on the observed scale because the absence of population prevalence data for the latent gSEM constructs precludes deriving values on the liability scale.

gSEM, genomic structural equation modeling; GWAS, genome-wide association study; SNP, single nucleotide polymorphism.

SZ liability was negatively correlated with that for *g*, as was BD liability, although less strongly. Despite the negative correlations with cognition, SZ liability was not associated with EA liability, but BD liability was associated with liability for higher EA. Similar estimates have been reported using a different methodology (27).

Shared liability also showed discordant effects, being negatively correlated with that for *g* but positively correlated with higher EA liability (0.07). In contrast, the SZ_diff_ fraction showed congruent effects, being negatively associated with liabilities to higher cognition and higher EA. It follows that the BD_diff_ fraction is correlated with liabilities to better cognition and higher EA.

### PRS Analyses

**Figure 2:**
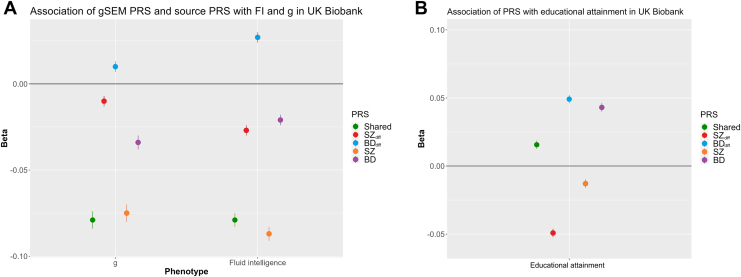
**(A)** Association of gSEM fractions and source GWAS PRSs with *g* and FI in the UK Biobank (FI *n* = 160,465; *g n* = 93,541). Point estimates for beta with standard errors. Note that the relative magnitudes of effects for different PRSs are not meaningful as they are dependent not only on the degree of shared genetic liability with the cognitive measures but also on the power of relevant input GWASs. The beta coefficient indicates the number of standard deviations that FI or *g* will increase or decrease by when the PRS increases by 1 SD. **(B)** Association of gSEM PRSs and source PRSs∗ with EA in the UK Biobank. Point estimates for beta with standard errors are given. Negative beta values indicate that higher liability to the relevant trait is associated with lower EA. Note that the relative magnitudes of effects for different PRSs are not meaningful as they are dependent not only on the degree of shared genetic liability with EA but also on the power of relevant input GWASs. BD, bipolar disorder; BD_d__iff_, BD differentiating fraction; EA, educational attainment; FI, fluid intelligence; *g*, general intelligence; gSEM, genomic structural equation modeling; GWAS, genome-wide association study; PRS, polygenic risk score; SZ, schizophrenia; SZ_d__iff_, SZ differentiating fraction.

The results of PRS analyses are provided in Figure 2A, B and in Tables S6 and S7.

The SZ, BD, and shared PRSs were negatively associated with *g* (SZ beta −0.075, *p* = 4.16 *×* 10^−51^; BD beta −0.035, *p* = 9.05 *×* 10^−17^; shared beta −0.079, *p* = 7.51 *×* 10^−51^). The SZ_diff_ fraction was weakly associated with lower *g* (SZ_diff_ beta −0.009, *p* = 3.74 *×* 10^−3^) and reciprocally BD_diff_ with higher *g*. The pattern of associations with FI was similar to those for *g* but with a stronger effect for the differentiating fraction (Figure 2A and Table S6).

The SZ PRS was associated with lower EA (beta −0.013, *p* = 2.46 *×* 10^−5^), while the BD PRS was associated with higher EA (beta 0.043, *p* = 9.07 *×* 10^−44^). Consistent with genetic correlation analysis, the shared PRS was associated with higher EA (beta 0.016, *p* = 5.08 *×* 10^−7^), while the SZ_diff_ fraction was associated with lower EA (beta −0.049, *p* = 1.84 *×* 10^−58^). Reciprocally, the BD_diff_ PRS was associated with higher EA.

### Cognitive and Noncognitive Effects on Education

Associations of shared liability with low cognition but higher EA suggest that it is enriched for alleles that promote EA through noncognitive mechanisms. In contrast, the concordant effects of SZ_diff_ on cognition and EA suggest that these alleles affect EA through effects on cognition. However, the effects of the SZ_diff_ PRS on EA covarying for cognition (primary test FI as we have more data and power than for *g*), while attenuated, remained significantly associated with poorer EA (unadjusted beta on a subset of UKBB participants with FI data: −0.042, SE = 0.005, *p* = 2.55 *×* 10^−17^; adjusted for FI: beta −0.025, SE = 0.005, *p* = 1.28 *×* 10^−6^), indicating that SZ_diff_ is enriched for alleles that have negative noncognitive as well as cognitive effects on EA.

### Enrichment Analyses

**Figure 3:**
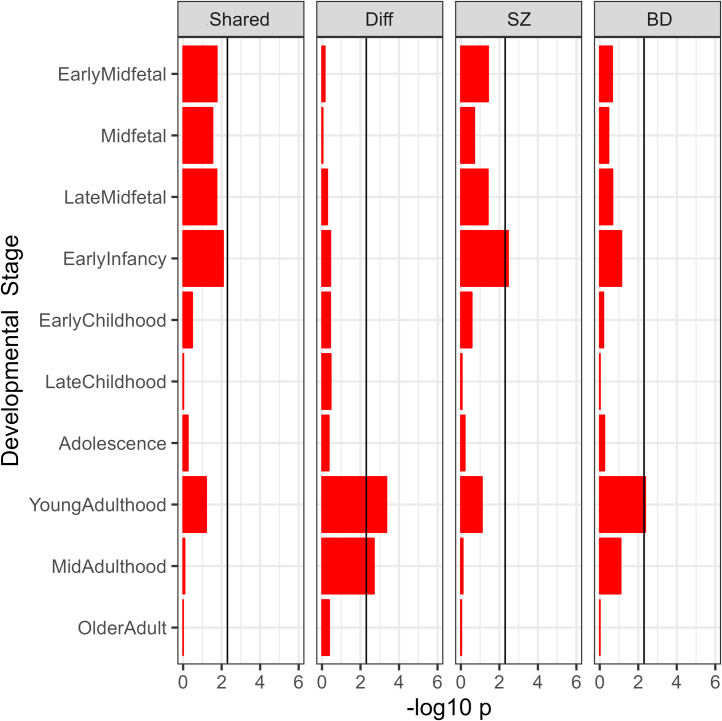
Enrichment of fractions of liability in genes with high specificity for developmental stages. −log10 *p* shows the significance level for stratified linkage disequilibrium score regression enrichment tests. The black line represents the corrected significance threshold (Bonferroni-corrected for 10 developmental stages). EarlyMidfetal samples are between pcw 10 and 16, Midfetal between pcw 16 and 17, LateMidfetal from between pcw 17 and 24, EarlyInfancy between birth and 6 months of age, EarlyChildhood between 1 and 6 years, LateChildhood between 6 and 13 years, Adolescence between 13 and 20 years, YoungAdulthood between 20 and 30 years, MidAdulthood between 30 and 60 years, and OlderAdult over 60 years. BD, bipolar disorder; pcw, postconception week; SZ, schizophrenia.

HES genes for young (ages 20–30 years) and mid- (ages 30–60) adulthood were significantly enriched for heritability that differentiates SZ from BD (Figure 3 and Table S8). In these two age groups, the HES genes only modestly overlap each other, as do the genes with evidence for association with the differentiating fraction, indicating that the enrichments at these stages are largely independent (Figure S1). BD showed stronger evidence for enrichment than SZ for heritability in these gene sets. SZ showed stronger evidence than BD for heritability enrichment in HES genes for early infancy, but this was not accompanied by enrichments in either gSEM fraction.

Details of the cellular heritability enrichments are provided in Figures S2 to S6 and Tables S9 to S13. Differentiating liability was not significantly enriched in HES genes for cell populations from human second trimester fetal brain (43), prefrontal cortex from gestation to adulthood (44,45), or from adult human prefrontal cortex and hippocampus (46). We did find significant enrichment of the differentiating fraction in HES genes for granule cells of the dentate gyrus in a more comprehensive dataset from adult human brain (47,48) (Figure S6 and Table S13). This set also showed significant evidence for enrichment in BD and nominally significant evidence in SZ and the shared liability fraction. In cell types from mouse brain (49) (Figure S3 and Table S10), heritability in the differentiating fraction was enriched for HES genes for pyramidal neurons from the somatosensory cortex and the CA1 region of the hippocampus and for medium spiny neurons of the striatum, but these findings were not replicated in the tested datasets from human brain (Figures S2 and S6). Moreover, these sets were also enriched for shared liability as well as liability to both source disorders.

GO enrichment analyses (Table S15) of differentiating liability identified no significant findings, while that of shared liability highlighted similar biological processes and molecular functions as the GWASs of SZ and of BD, although more categories were significant (58 in shared, 38 in SZ, 11 in BD).

## Discussion

Our findings are consistent with our primary hypothesis that alleles that preferentially increase liability to SZ over BD are associated with lower cognitive performance in the general population, whereas genetic liability that increases liability to BD over SZ is associated with higher performance. We also showed that the fraction shared between the 2 disorders was associated with poorer cognition, consistent with observations that both disorders are associated with cognitive impairment. The opposing effects of the differentiating fractions provide a partial explanation for the greater cognitive impairments in SZ compared with BD (30,53), but their relatively modest effects are also consistent with evidence that nonfamilial factors, such as environmental exposures and de novo mutations rather than familial ones (including inherited genetic variation), are the main cause of cognitive impairments in SZ (29). Together with evidence that nonfamilial factors play a greater role in SZ than in BD (54), our findings support the hypothesis that these are more important than common genetic variation in the greater cognitive impairment seen in SZ than BD.

To the best of our knowledge, ours is the first study to compare the relationships between shared and specific fractions of genetic liability to SZ and BD with direct measures of cognitive function. However, our results are consistent with findings from studies that used different methods to compare genetic liability to SZ with that for BD. These include a study (55) that found that most alleles shared by SZ and *g* were associated with poorer cognition, whereas most BD alleles shared with *g* were associated with better cognition. Another study (27) using a bivariate causal mixture model showed high overlap between variant sites that influence *g* and those that confer liability to BD and SZ; however, like us, they found low to moderate genetic correlations. Extensive overlapping sites but modest genetic correlations implies that risk alleles to the psychiatric disorders include a mixture of alleles associated with higher and lower intelligence. Additional analyses using LAVA (56) also showed prominent mixed directions of effect between BD, SZ, and cognitive traits.

Our secondary aim was to examine the relationships between fractions of liabilities to SZ and BD and liability to EA and to measured EA. Shared liability was weakly but significantly correlated with liability to higher EA (Figure 1) and higher measured EA (Figure 2B), while the SZ_diff_ fraction was negatively correlated with liability to higher EA but was associated with lower measured EA. Therefore, SZ liability includes a greater proportion of risk alleles that negatively influence EA than liability to BD, which may explain why despite the high genetic correlation between the 2 disorders, we found that liability to BD was associated with better EA and liability to SZ with poorer EA.

Our study also extends work on the relationships between the cognitive and noncognitive components of EA and the shared and specific fractions of liability to SZ and BD (21,57,58) by incorporating direct measures of cognition and of EA. The counterintuitive observation that while shared liability is associated with poorer cognition (Figure 2A), it is also associated with higher EA (Figure 2B), implies that the effects of shared liability on EA comes from alleles associated with noncognitive traits that promote higher EA. In contrast, the observations that SZ_diff_ is associated with poorer cognition and with poorer EA and that the association with EA is attenuated after conditioning on cognitive ability suggest that this fraction of liability exerts effects on EA through cognitive mechanisms. However, this association was only partially attenuated, suggesting that SZ_diff_ also exerts effects on noncognitive traits that promote lower EA. Nevertheless, given that overall liability to SZ shows little association with liability to or measured EA performance, the opposing effects of alleles from the shared and SZ_diff_ fractions must largely cancel each other out. These findings have important implications for interventions designed to improve educational outcomes in SZ, which we suggest may need to focus on noncognitive as well as cognitive mechanisms. They also suggest that there are important shortcomings associated with using EA in genomic studies as a proxy for cognitive function.

Our finding that in the general population, genetic liability to SZ conferred by common heritable alleles was associated with better EA than expected given their effects on cognitive ability (Figure 2A, B) is surprising given that overall risk of the disorder is associated with poorer EA (59). However, it is consistent with evidence that SZ is more strongly associated with the extent to which EA in people deviates from that of their family members and that this deviation is not explained by heritable liability to SZ (60).

SZ is more strongly associated than BD is with cognitive impairment, leading us to predict that differentiating liability would be enriched for HES genes for prenatal and early childhood developmental stages and cells of the developing brain, but this was not observed. This is consistent with the hypothesis that nonfamilial factors play a larger role than common genetic variation in the greater neurodevelopmental impairment seen in SZ than BD. Contrary to our expectation, differentiating liability was enriched in HES genes for early and mid-adulthood, an age range likely to index later, rather than early, neurodevelopmental processes. However, this stage of development corresponds to the typical age at onset of psychotic symptoms, the severity of which was reported to be associated with the SZ_diff_ fraction in people with BD (7). Using cell-specific gene expression data from adult human brain, we also observed an enrichment of the differentiating fraction in HES genes for granule cells of the adult dentate gyrus, the function of which has been proposed as central to the genesis of psychotic symptoms (61). The same set of HES genes was also more strongly enriched in the GWAS for BD than in the GWAS for SZ (Figure S6). Therefore, studies of the dentate gyrus and the relevant associated genes may offer a window into biology that is potentially more important for BD; in fact, a hyperexcitable phenotype has been reported in induced pluripotent stem cell–derived granule neurons from people with BD (62).

### Strengths and Limitations

We studied cognition in individuals without severe mental illness to reduce the impact of medication effects and reverse causation. We used both FI and a measure of *g*, which we formed from a principal component analysis of 4 other cognitive tests. We chose these to ensure that our findings went beyond the analysis of a single cognitive measure. *g* also gives a more robust measure of general cognitive ability (63,64), and psychotic disorders are associated with broad, multidomain cognitive impairments (14), including in *g*. Moreover, the source GWASs showed associations with cognitive function that were consistent with expectations based on the degrees of cognitive impairment seen in the 2 disorders and in previous correlational studies between the disorders and intelligence. This reassures us that the cognitive measures that we used were comparable to those used in previous studies that demonstrated impaired cognitive function in these disorders. In addition, our results were consistent across the 2 measures of cognition that we used. Our interpretation that the discordant findings between effects of liability on measures of cognition and EA point to effects on noncognitive traits that influence EA is that these findings could be explained by aspects of cognition that are not captured by *g* or FI. Individuals in the UKBB differ from individuals in the general population, and in particular they have higher than average levels of EA and cognitive function (23), which may result in underestimation of the effect sizes of associations with these traits. In addition, the single-nucleus RNA sequencing datasets from human postmortem brain that we tested in this study are likely to underrepresent synaptic genes (49), which are known to be relevant to psychiatric disorders (30,31).

### Conclusions

Liability that is shared between SZ and BD is enriched for alleles that confer risk for poorer cognitive function in the general population but is associated with noncognitive traits that enhance EA. In contrast, SZ_diff_ is enriched for alleles that confer risk for poorer EA through both cognitive and noncognitive mechanisms. Establishing the relevant noncognitive traits may afford opportunities for intervention. Alleles that differentiate between SZ and BD are enriched for genes with HES for early and mid-adulthood and for granule cells of the dentate gyrus. Follow-up studies focusing on genes with HES for these time points and brain region may provide insights into the biology that distinguishes these two major psychiatric disorders.

## Supplementary Material

Tables S1–S15

Key Resources Table
