# Supplementary material for: Effects of Shared and Nonshared Schizophrenia and Bipolar Disorder Alleles on Cognition and Educational Attainment in the UK Biobank
Source: Biol Psychiatry Glob Open Sci. 2025 Aug 22;5(6):100601. doi: 10.1016/j.bpsgos.2025.100601 (PMC12516558; doi:10.1016/j.bpsgos.2025.100601)
Supplement: Supplemental Methods and Figures S1–S6 [file mmc1.pdf]

SUPPLEMENTARY INFORMATION

Effects of Shared and Nonshared Schizophrenia and Bipolar Disorder Alleles on Cognition and Educational Attainment in the UK Biobank

Richards *et al.*

Supplementary Methods

Genomic SEM

The GWAS summary statistics for gSEM were from studies of SZ and BD conducted by the Psychiatric Genomics Consortium (PGC)[1,2] (Supplementary Table S1). The BD GWAS included only participants of European ancestry, so we similarly restricted the SZ GWAS summary statistics to those of European ancestry to avoid ancestry related differences in allele frequency and patterns of linkage disequilibrium confounding our estimates of genomic differences between the disorders[3].

Single nucleotide polymorphisms (SNPs) were included in the analyses if they had a minor allele frequency greater than 1% in HapMap 3 reference set[4] , were present in both source GWASs, and had an imputation score of at least 0.7. Variants within the extended MHC were excluded (chromosome 6; 25-35 Mb) as it is difficult to allow for the complex LD structure in the region. We retained 7,334,582 SNPs for analysis. We used gSEM to apply a common factor model to the summary statistics from the GWAS (see 'lavaan code for two models' below). gSEM was run in R (The R Foundation, version 4.0.3) using the GenomicSEM package[5]. gSEM estimates and corrects for sample overlap among the input GWAS[6]. For each SNP, the loading on the common factor was extracted to produce a statistic corresponding to the effect of that variant that is shared between SZ and BD. We then applied a model where we extracted the loading of each SNP on the residual variance from each input GWAS that was not explained by the common factor (see see 'lavaan code for two models' below) so that the residual effect sizes for each SNP indexes how much it influences the probability of having one phenotype over the other. The SZ differentiating fraction (SZ_{diff}) denotes effects signed such that beta above zero indicates an allele that increases the probability of SZ over BD, while those below zero indicate the reverse. Conversely, we refer to the bipolar differentiating fraction (BD_{diff}) to denote effects signed such that beta above zero indicates an allele that increases the probability of BD over SZ, while those below zero indicate the reverse. Note that in the present study, as there are only two phenotypes in the model, SZ_{diff} and BD_{diff} are perfectly negatively correlated.

SNP-based heritabilities (SNPh2) and genetic correlations were calculated using linkage disequilibrium score regression[6,7]. We report heritability on the observed scale because the population prevalences of the latent constructs underlying gSEM fractions are unknown, precluding conversion to a liability scale.

lavaan code for two models

<u>Shared model</u>

F1 =~ a*SZ + a*BD

F1 ~ SNP

F1 ~~ 1*F1

This model creates a shared fraction (F1) and examines its effect on each SNP. The number of parameters, including the output of interest, that gSEM can estimate is limited by the number of inputs, here the SZ GWAS and BD GWAS. To make the model identifiable and possible to run, it is necessary to constrain the number of parameters the model needs to estimate. The model code achieves this by loading each source GWAS onto the shared component equally (line 1 of code) and constraining the variance of the shared fraction to 1 (line 3 of code).

<u>Differentiating model</u>

```
F1 =~ SZ + BD

F2 =~ SZ

F1 ~ SNP

F2 ~ SNP

F1 ~~ F1

F2 ~~ F2

F1 ~~ 0*F2

SZ ~~ 0*SZ

BD ~~ 0*BD

SZ ~~ 0*BD
```

This model creates a shared fraction (F1) from the SZ and BD source GWAS, and an F2 fraction that captures the residual variance not captured by the shared component (lines 1 and 2). The effect of these fractions on each SNP are assessed (lines 3 and 4). The F1 and F2 components have their variance constrained to 1 to make the model identifiable (lines 5 and 6). They are minimally correlated with each other (line 7), and have their residual variance and covariance set to 0 (lines 8-10).

Genotyping in UK Biobank

Exclusion criteria for genetic variants in the UK Biobank data were: genotyping rate < 0.95, minor allele frequency < 0.01, Hardy-Weinberg equilibrium (HWE) p-value < 1e-6 (using the ‘midp’ and ‘keep-fewhet’ options), imputation INFO score < 0.9. Individuals were excluded if they had SNP missingness > 0.05.

Phenotyping in UK Biobank

We excluded people with a diagnosis of bipolar disorder, schizophrenia or a psychotic disorder based on primary care data, hospital inpatient data, death register records, or self-report.[8]

Cognitive assessments in the UK Biobank were brief and unsupervised. Individual cognitive test scores from this dataset vary in reliability and stability[9,10], but performance between different cognitive tests is correlated[10,11] which enables a more robust measure of generalised cognition (*g*) to be derived from a principal component analysis of multiple cognitive tests[9]. Our approach to deriving a measure of *g* and the rationale for the inclusion/exclusion of individual cognitive tests are provided elsewhere[12.] Briefly, *g* was derived as the first principal component from analysis of: numeric memory (from the first online cognitive test battery); reaction time (from baseline assessment); pairs matching (from baseline assessment); and trail making test B (from the first online cognitive test battery). *g* was only available for participants who had completed all four tests (n = 94956, Supplementary Tables S2). *g* was standardised and outliers were removed (*g* score > 4 or < -4). The UK Biobank also has data available on a subset of participants for a measure of fluid intelligence (FI). FI was measured at either the initial assessment or one of two subsequent assessments. FI was standardised, then if it was measured at multiple timepoints, we took the mean value. FI was not included in our estimate of *g*, allowing us to test the relationship between the gSEM fractions and FI as a check of robustness of our findings in relation to *g.*

The Biobank data included a variable representing level of education achieved by participants. As in our previous publication[13], we used these data to derive an ordinal measure representing the highest educational level the participant had achieved (1=no qualifications, 2=NVQ/HND/HNC or equivalent, 3=CSE or equivalent, 4=O-level/GCSE or equivalent, 5=A/AS-levels or equivalent, 6=college or university degree). Professional qualifications were not considered, as the level of academic achievement they represent varies by profession.

Samples with both genotype and phenotype data were aged between 39 and 71 (mean age 56.6 years) and were 54% female, 46% male.

Polygenic Risk Score (PRS) Analyses

PRS were derived as described[14]. Clumping was performed on imputed best-estimate genotypes using PLINK (maximum r2 = 0.2; window = 500 kb; minimum minor allele frequency = 0.1; minimum info score = 0.7). Variants within the extended MHC were excluded (chromosome 6 from 25 MB to 35 MB). As optimal P value thresholds for inclusion of alleles in the gSEM-derived PRS are unknown and no large independent samples are available to derive them, we performed PRS analysis without P value thresholding[3].

Association tests were adjusted for the first 10 population principal components, sex, age at interview, age at interview squared and genotyping platform. Educational attainment analyses were adjusted for birth before 1950 to reflect changes in the educational grading system in the UK[15]. All PRS variables and the fluid intelligence and *g* variables were standardized before analysis using the scale() function in R. We tested PRS for association with *g* (93541 participants, Supplementary Table S3) and FI (160465 participants) using linear regression, reporting beta and P values for the PRS term in the regression

model. PRS were tested for association with the ordinal measure corresponding to the highest educational level achieved using ordinal regression (354609 participants). All P values were 2-tailed.

Developmental Stage Enrichment Analyses

Transcriptomic data from the human dorsolateral prefrontal cortex (DLPFC) and hippocampus were obtained from the BrainSeq Phase II database[16]. This consisted of 300 DLPFC and 314 hippocampal samples from 374 individuals, ranging from 12 post-conception weeks to 84 years (further details in Supplementary Table S4). Samples were divided into developmental stages defined in Supplementary Table S5. Raw read counts were normalized using the trimmed mean of M-values (TMM) method[17]. For each gene, expression values were regressed against a single developmental stage, modelled as a binary variable indicating whether a sample belonged to the stage under consideration or not. Brain region, sex, and genetic ancestry, represented by the first five principal components of genotype, were included as covariates, and within-individual correlation was accounted for using the duplicateCorrelation function in the limma R package. From each model, the t-statistic for the developmental stage term was extracted for every gene, serving as a measure of developmental stage expression specificity relative to all other stages[18,19]. The top 10% stage-specific genes, ranked by their t-statistics, were selected to define stage-specific gene sets. These gene sets were then tested for enrichment of associations in the gSEM shared and differentiating fractions using stratified LD score regression (SLDSR) v1.2[6,20]. Use of SLDSR on genes in the top decile of expression specificity has been shown to have statistical power and low false discovery rates that are superior or equivalent to other methods for identifying relevant tissues[21]. We followed Finucane et al. (2018) in applying a 100 kb window around each gene and incorporating version 1.2 of the baseline LD annotations[20]. The one-sided coefficient z-score p-value was extracted as a measure of significance in the gSEM shared and differentiating fractions in each gene set. Enrichment analyses were also performed on the SZ and BD source GWAS datasets for comparison.

Cellular Enrichment Analyses

Cellular gene expression specificity scores were obtained from five independent datasets: four single-nucleus RNA-Seq datasets from human brain and one single-cell RNA-Seq study from mouse. These included 91 cell populations across five regions of the human fetal brain[22], 84 populations from the human prefrontal cortex spanning gestation to adulthood[23,24], 15 populations from the adult human frontal cortex and hippocampus [25] 31 'higher-order' cell superclusters of the adult human brain[21,26] and 24 populations from the mouse brain[47.] Further details are provided in Supplementary Table S5. Gene expression specificity scores were calculated by dividing each gene's normalized unique molecular identifier (UMI) count in a given cell type by the sum of that gene's expression across all cell types. Genes mapping to the extended MHC region or chromosome X were excluded. The top 10% of genes with the highest specificity scores for each cell type were tested for enrichment of associations in the gSEM shared and differentiating fractions using stratified LD score regression v1.2[6,20], as described above, with the SZ and BD source GWAS datasets again tested for comparison.

Gene Ontology Enrichment Analyses

We tested for enrichment of associations in the GSEM shared and differentiating fractions, as well as source SZ and BD GWAS datasets, in Gene Ontology (GO) terms using MAGMA (v1.10)[49]. GO terms were downloaded from the Gene Ontology Consortium[27] (geneontology.org, November 9, 2020). Low-confidence gene-category relationships, such as those inferred from electronic annotation (IEA), non-traceable author statements (NAS), or reviewed computational analysis (RCA), were excluded, along with obsolete categories[28]. For the MAGMA analyses, SNP-level p-values were aggregated into gene-level statistics using a snp-wise=mean model, with a 35 kb upstream and 10 kb downstream window applied to each gene's transcribed sequence. One-sided competitive p-values for each GO term were extracted as the primary test statistics.

References

1. Mullins N, Forstner AJ, O'Connell KS, et al. Genome-wide association study of more than 40,000 bipolar disorder cases provides new insights into the underlying biology. *Nat Genet*. 2021;53(6):817-829. doi:10.1038/s41588-021-00857-4

2. Trubetskoy V, Pardiñas AF, Qi T, et al. Mapping genomic loci implicates genes and synaptic biology in schizophrenia. *Nature*. 2022;604(7906):502-508. doi:10.1038/s41586-022-04434-5

3. Richards AL, Cardno A, Harold G, et al. Genetic liabilities differentiating bipolar disorder, schizophrenia, and major depressive disorder, and phenotypic heterogeneity in bipolar disorder. *JAMA Psychiatry*. 2022;79(10):1032-1039. doi:10.1001/jamapsychiatry.2022.2594

4. International HapMap 3 Consortium, Altshuler DM, Gibbs RA, et al. Integrating common and rare genetic variation in diverse human populations. *Nature*. 2010;467(7311):52-58. doi:10.1038/nature09298

5. Grotzinger AD, Rhemtulla M, de Vlaming R, et al. Genomic structural equation modelling provides insights into the multivariate genetic architecture of complex traits. *Nat Hum Behav*. 2019;3(5):513-525. doi:10.1038/s41562-019-0566-x

6. Bulik-Sullivan B, Loh P-R, Finucane HK, et al. LD Score regression distinguishes confounding from polygenicity in genome-wide association studies. *Nat Genet*. 2015;47(3):291-295. doi:10.1038/ng.3211

7. Bulik-Sullivan B, Finucane HK, Anttila V, et al. An atlas of genetic correlations across human diseases and traits. *Nat Genet*. 2015;47(11):1236-1241. doi:10.1038/ng.3406

8. Legge SE, Pardiñas AF, Woolway G, et al. Genetic and phenotypic features of schizophrenia in the UK biobank. *JAMA Psychiatry*. 2024;81(7):681-690. doi:10.1001/jamapsychiatry.2024.0200

9. Fawns-Ritchie C, Deary IJ. Reliability and validity of the UK Biobank cognitive tests. *PLoS ONE*. 2020;15(4):e0231627. doi:10.1371/journal.pone.0231627

10. Lyall DM, Cullen B, Allerhand M, et al. Cognitive test scores in UK biobank: data reduction in 480,416 participants and longitudinal stability in 20,346 participants. *PLoS ONE*. 2016;11(4):e0154222. doi:10.1371/journal.pone.0154222

11. Spearman C. “general intelligence” objectively determined and measured. In: Jenkins JJ, Paterson DG, eds. *Studies in Individual Differences: The Search for Intelligence.* Appleton-Century-Crofts; 1961:59-73. doi:10.1037/11491-006

12. Fenner E, Holmans P, O’Donovan MC, Owen MJ, Walters JT, Rees E. Rare coding variants in schizophrenia-associated genes affect generalised cognition in the UK Biobank. *medRxiv*. August 16, 2023. doi:10.1101/2023.08.14.23294074

13. Escott-Price V, Bracher-Smith M, Menzies G, et al. Genetic liability to schizophrenia is negatively associated with educational attainment in UK Biobank. *Mol Psychiatry*. 2020;25(4):703-705. doi:10.1038/s41380-018-0328-6

14. International Schizophrenia Consortium, Purcell SM, Wray NR, et al. Common polygenic variation contributes to risk of schizophrenia and bipolar disorder. *Nature*. 2009;460(7256):748-752. doi:10.1038/nature08185

15. Leonenko G, Di Florio A, Allardyce J, et al. A data-driven investigation of relationships between bipolar psychotic symptoms and schizophrenia genome-wide significant genetic loci. *Am J Med Genet B Neuropsychiatr Genet*. 2018;177(4):468-475. doi:10.1002/ajmg.b.32635

16. Collado-Torres L, Burke EE, Peterson A, et al. Regional Heterogeneity in Gene Expression, Regulation, and Coherence in the Frontal Cortex and Hippocampus across Development and Schizophrenia. *Neuron*. 2019;103(2):203-216.e8. doi:10.1016/j.neuron.2019.05.013

17. Robinson MD, McCarthy DJ, Smyth GK. edgeR: a Bioconductor package for differential expression analysis of digital gene expression data. *Bioinformatics*. 2010;26(1):139-140. doi:10.1093/bioinformatics/btp616

18. Clifton NE, Hannon E, Harwood JC, et al. Dynamic expression of genes associated with schizophrenia and bipolar disorder across development. *Transl Psychiatry*. 2019;9(1):74. doi:10.1038/s41398-019-0405-x

19. Clifton NE, Collado-Torres L, Burke EE, et al. Developmental Profile of Psychiatric Risk Associated With Voltage-Gated Cation Channel Activity. *Biol Psychiatry*. 2021;90(6):399-408. doi:10.1016/j.biopsych.2021.03.009

20. Finucane HK, Reshef YA, Anttila V, et al. Heritability enrichment of specifically expressed genes identifies disease-relevant tissues and cell types. *Nat Genet*. 2018;50(4):621-629. doi:10.1038/s41588-018-0081-4

21. Yao S, Harder A, Darki F, et al. Connecting genomic results for psychiatric disorders to human brain cell types and regions reveals convergence with functional connectivity. *Nat Commun*. 2025;16(1):395. doi:10.1038/s41467-024-55611-1

22. Cameron D, Mi D, Vinh N-N, et al. Single-Nuclei RNA Sequencing of 5 Regions of the Human Prenatal Brain Implicates Developing Neuron Populations in Genetic Risk for Schizophrenia. *Biol Psychiatry*. 2023;93(2):157-166. doi:10.1016/j.biopsych.2022.06.033

23. Herring CA, Simmons RK, Freytag S, et al. Human prefrontal cortex gene regulatory dynamics from gestation to adulthood at single-cell resolution. *Cell*. 2022;185(23):4428-4447.e28. doi:10.1016/j.cell.2022.09.039

24. Tume CE, Chick SL, Holmans PA, et al. Genetic implication of specific glutamatergic neurons of the prefrontal cortex in the pathophysiology of schizophrenia. *Biological Psychiatry Global Open Science*. 2024;4(5):100345. doi:10.1016/j.bpsgos.2024.100345

25. Habib N, Avraham-Davidi I, Basu A, et al. Massively parallel single-nucleus RNA-seq with DroNc-seq. *Nat Methods*. 2017;14(10):955-958. doi:10.1038/nmeth.4407

26. Siletti K, Hodge R, Mossi Albiach A, et al. Transcriptomic diversity of cell types across the adult human brain. *Science*. 2023;382(6667):eadd7046. doi:10.1126/science.add7046

27. Ashburner M, Ball CA, Blake JA, et al. Gene Ontology: Tool for the unification of biology. *Nat Genet*. 2000;25(1):25-29. doi:10.1038/75556

28. Gene Ontology Consortium, Aleksander SA, Balhoff J, et al. The Gene Ontology knowledgebase in 2023. *Genetics*. 2023;224(1):iyad031. doi:10.1093/genetics/iyad031

Supplementary Figures

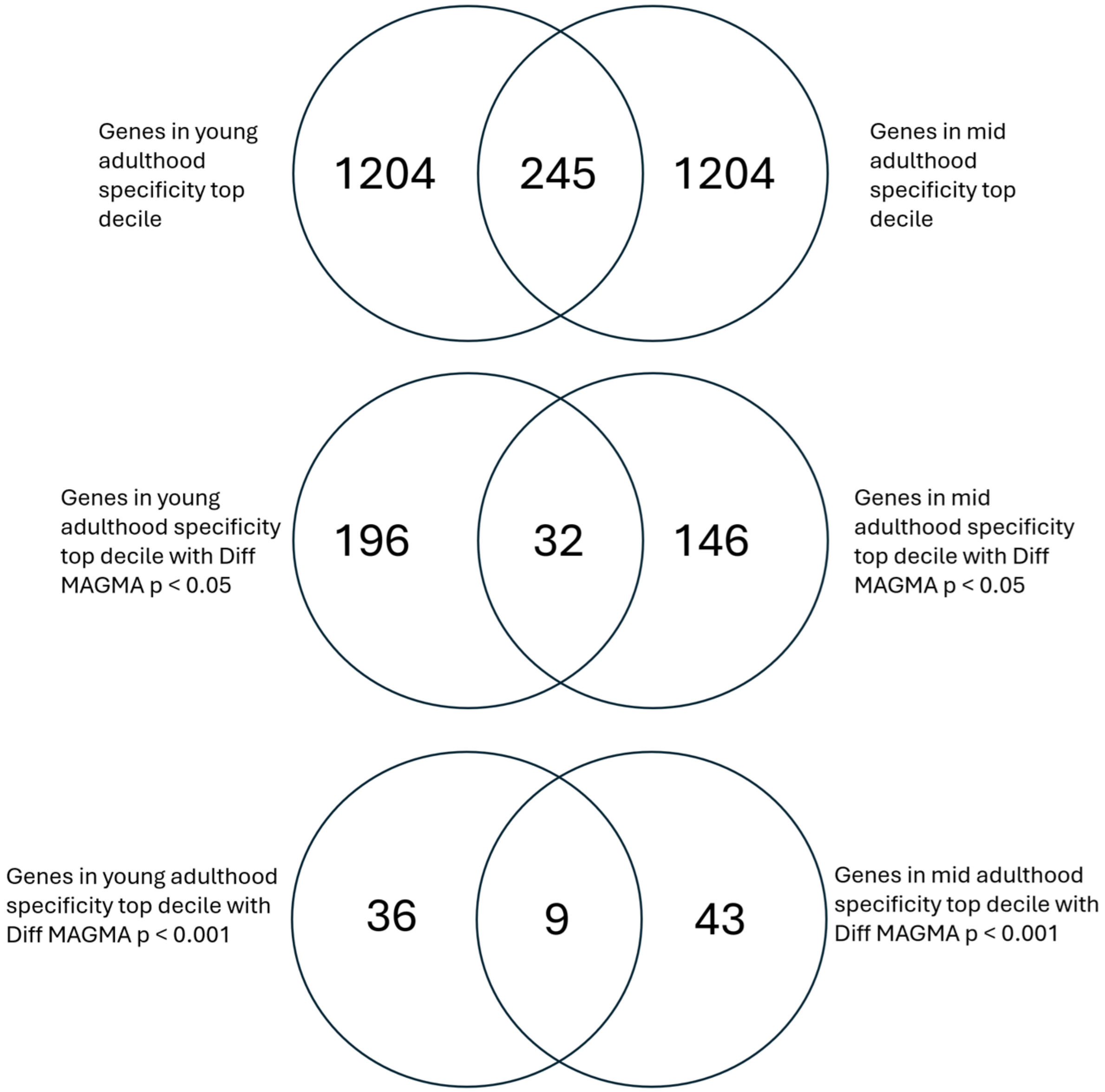

Figure S1. Venn diagram showing overlap between genes in top deciles of specificity for young adulthood and mid adulthood. MAGMA gene p-values given in Supplementary Table S14.

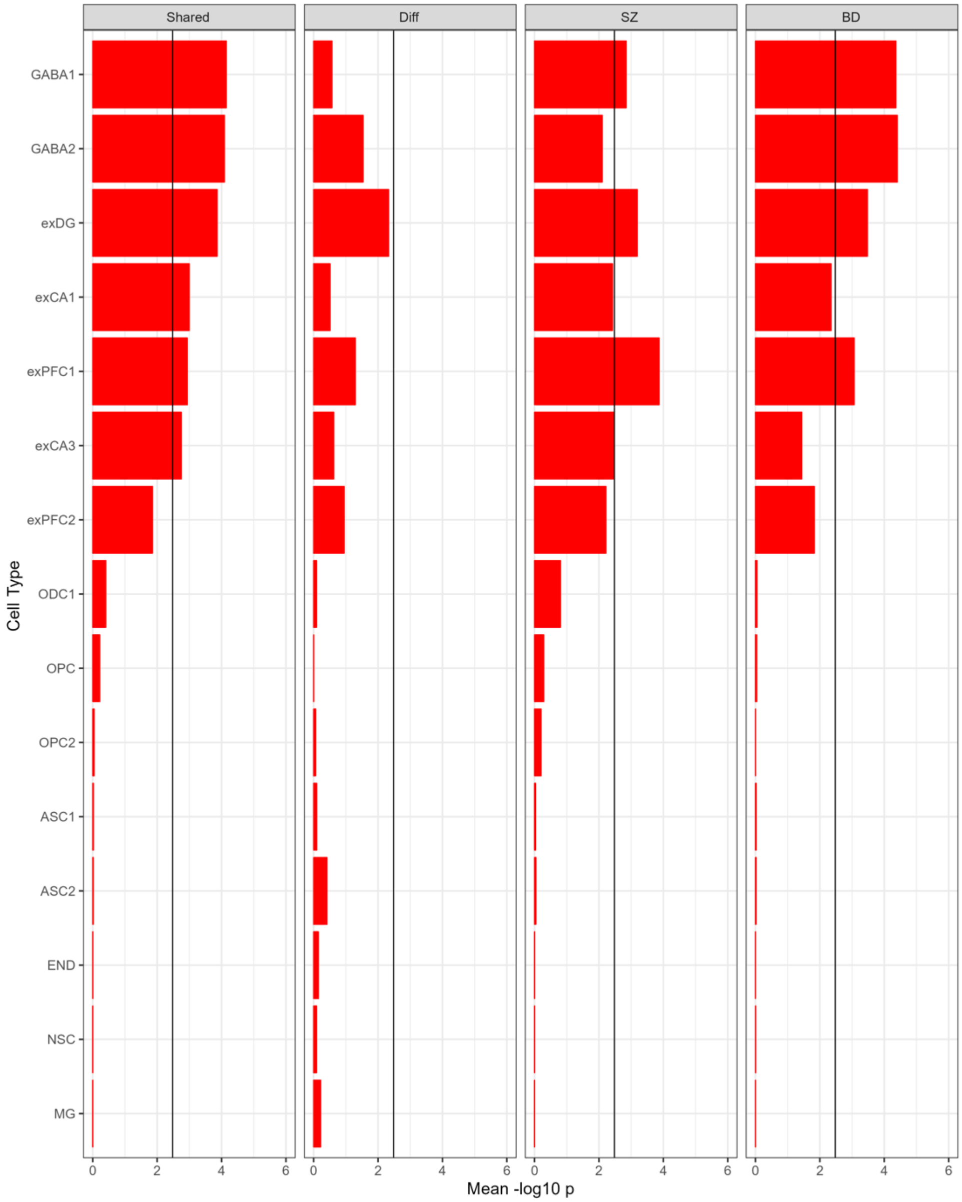

Figure S2. Enrichment of genomic SEM SZ/BD shared and differentiating components and SZ and BD GWAS in adult human prefrontal cortex and hippocampus using stratified LDSR[1,2]. Gene sets tested for enrichment comprise genes with the top 10% expression specificity values for cell types in the adult hippocampus and prefrontal cortex single nucleus RNA sequencing dataset generated by Habib et al[3]. Gene expression specificity values calculated by Skene et al[4]; cell populations are labeled in accordance with Habib et al[3]. Black line represents significance threshold (Bonferroni corrected for 15 cell types).

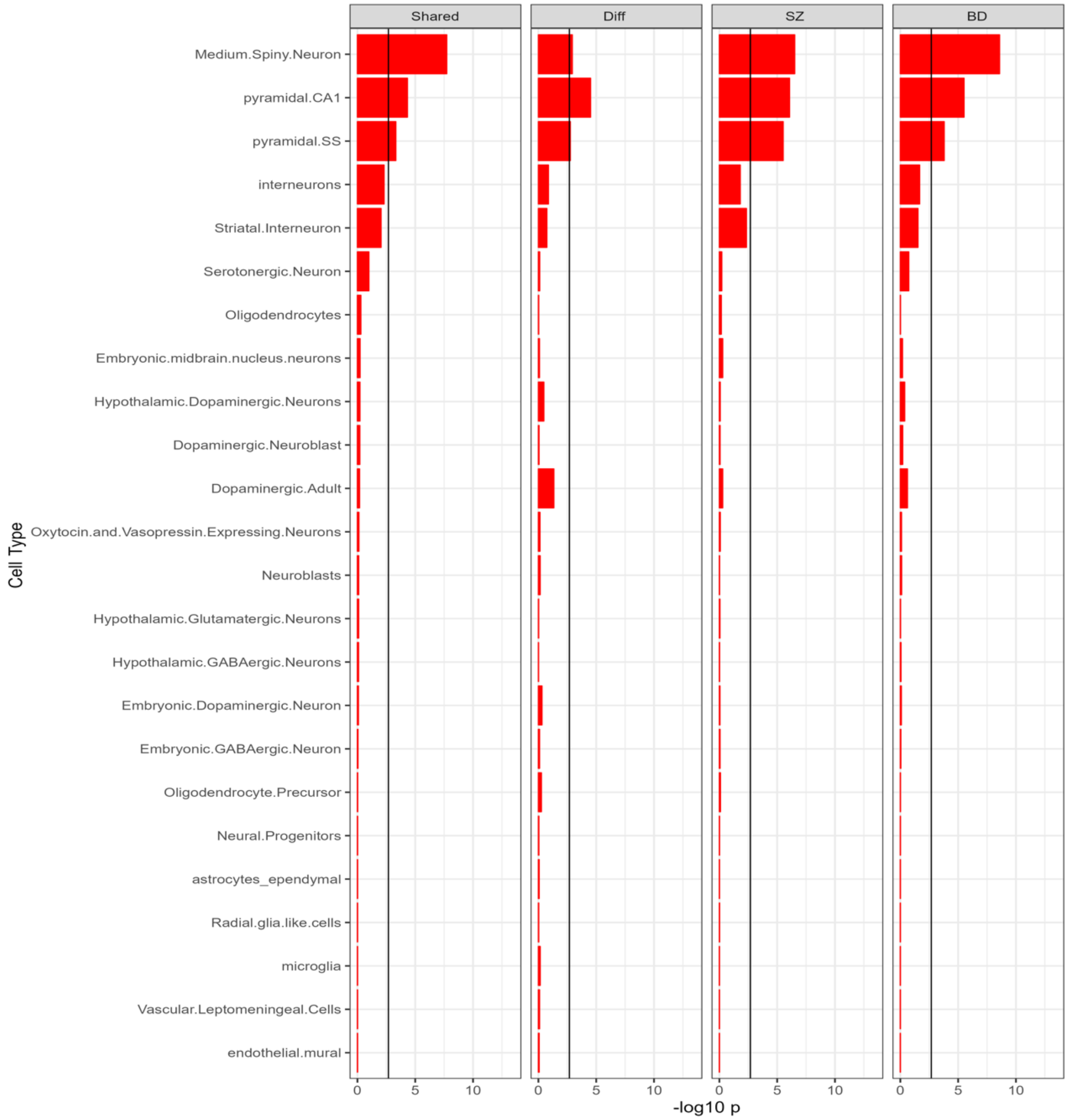

Figure S3. Enrichment of genomic SEM SZ/BD shared and differentiating components and SZ and BD GWAS in mouse brain and cell populations using stratified LDSR[1,2]. Gene sets tested for enrichment comprise genes with the top 10% expression specificity values in cell types of the mouse brain (generated by single cell RNA sequencing) as calculated by Skene et al[4]. Cell populations are labeled in accordance with Skene et al[4]. Black line represents significance threshold (Bonferroni corrected for 24 cell types).

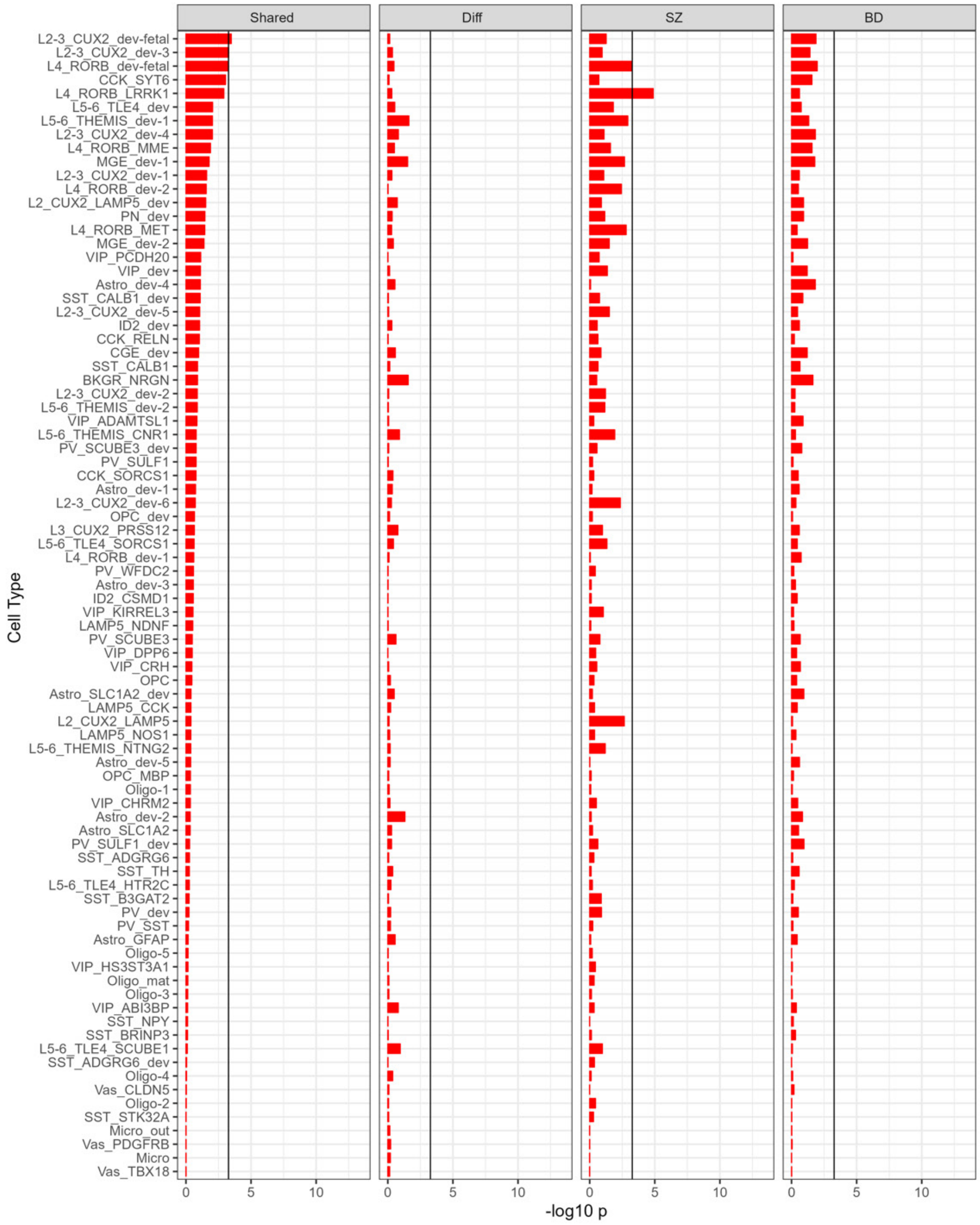

Figure S4. Enrichment of genomic SEM SZ/BD shared and differentiating components and SZ and BD GWAS in cell populations of the human prefrontal cortex using stratified LDSR[1,2]. Gene sets tested for enrichment comprise genes with the top 10% expression specificity values for cell populations of the human prefrontal cortex from gestation to adulthood, as calculated by Tume *et al*[5] based on single nucleus RNA sequencing from the study of Herring *et al*[6]. Cell populations are labeled in accordance with Herring et al[6]. Black line represents significance threshold (Bonferroni corrected for 84 cell types).

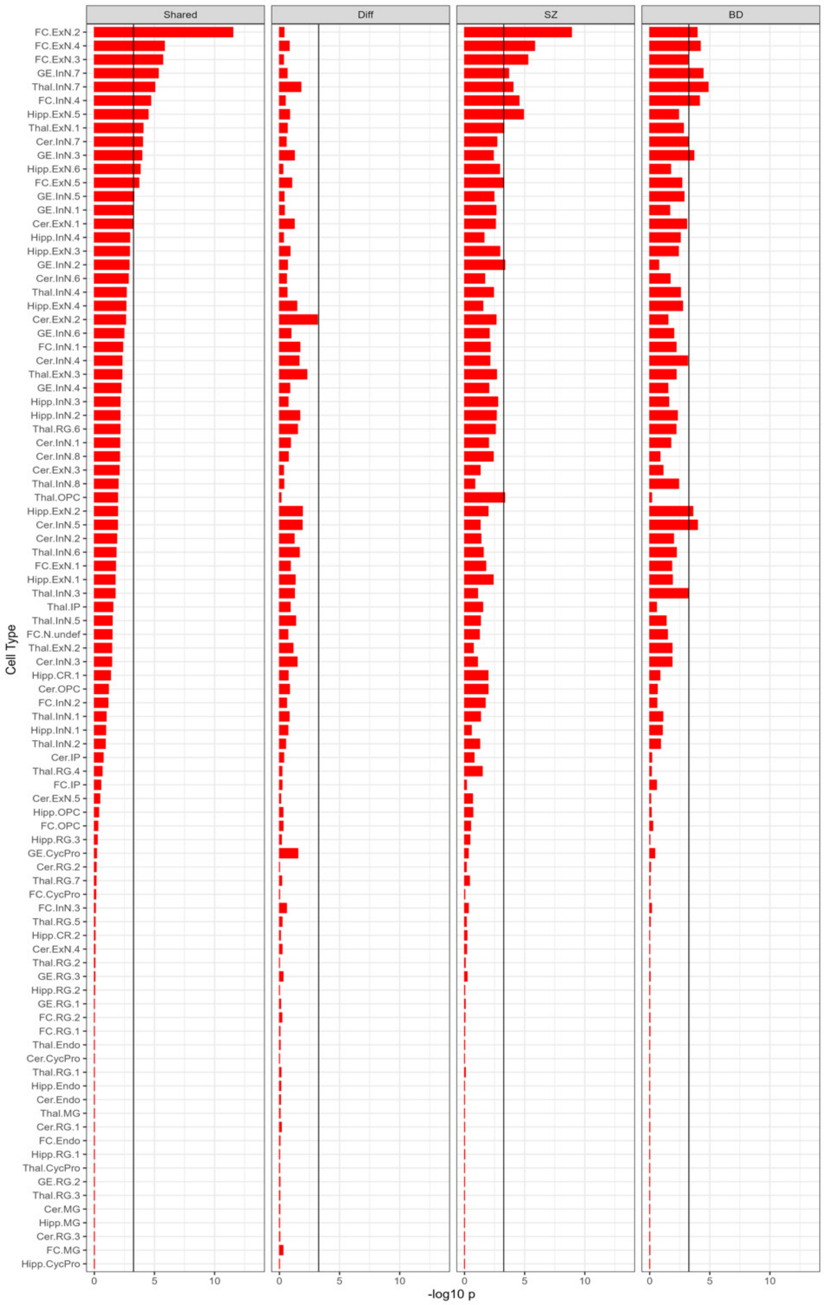

Figure S5. Enrichment of genomic SEM SZ/BD shared and differentiating components and SZ and BD GWAS in cells of the human fetal brain using stratified LDSR[1,2]. Gene sets tested for enrichment comprise genes with the top 10% expression specificity values for cell populations in 5 regions of the human second trimester fetal brain generated by Cameron *et al*[7] using single nucleus RNA sequencing. Cell populations are labeled in accordance with Cameron *et al*[7]. Black line represents significance threshold (Bonferroni corrected for 91 cell types across all five brain areas).

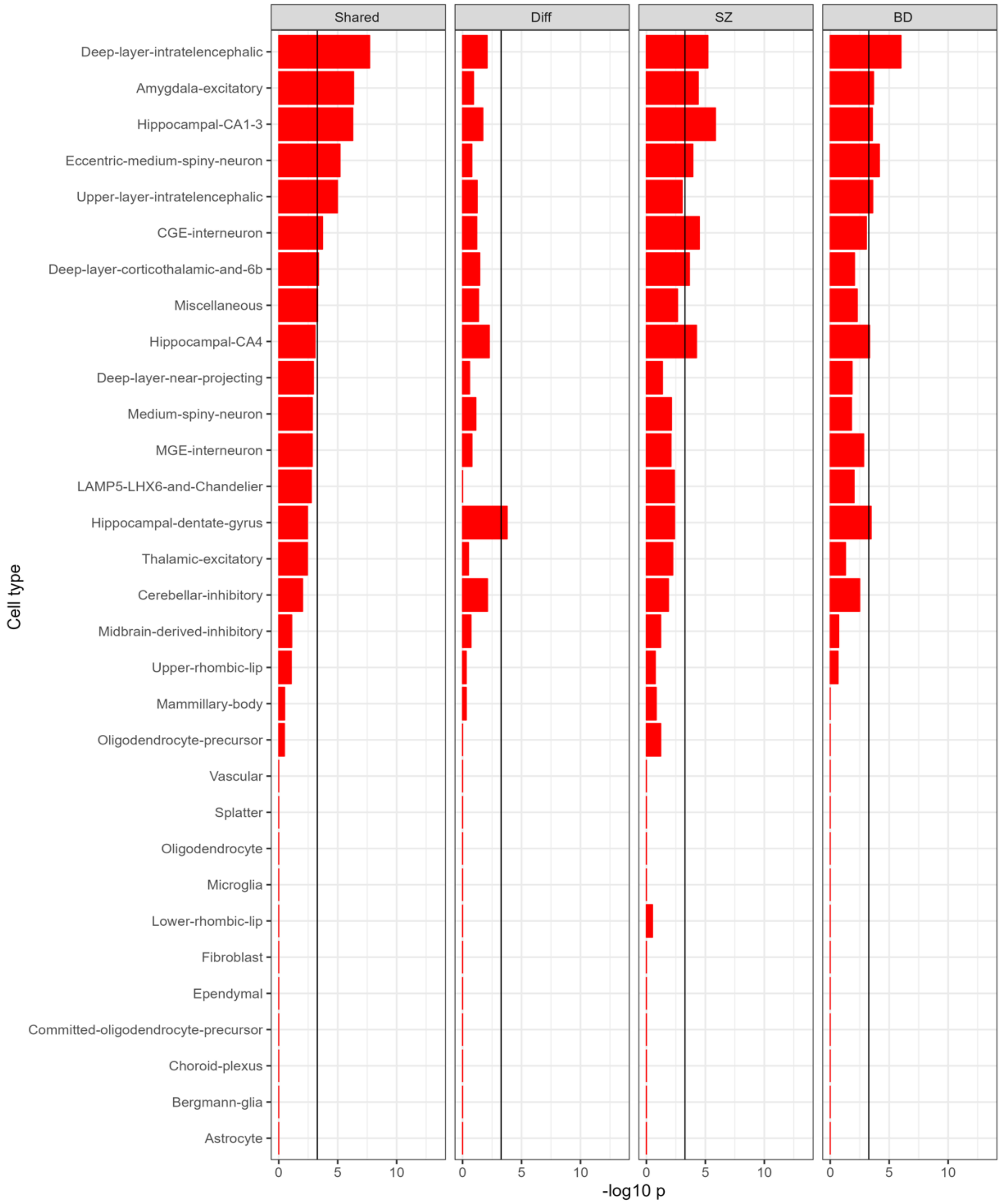

Figure S6. Enrichment of genomic SEM SZ/BD shared and differentiating components and SZ and BD GWAS in cells of the adult human brain using stratified LDSR[1,2]. Gene sets tested for enrichment comprise genes with the top 10% expression specificity values for 31 'higher-order' cell superclusters of the adult human brain calculated by Yao *et al*[8] based on single nucleus RNA sequencing by Siletti *et al*[9]. Cell populations are labeled in accordance with Siletti *et al*[9]. Black line represents significance threshold (Bonferroni corrected for 31 cell populations).

References

1. Finucane HK, Reshef YA, Anttila V, et al. Heritability enrichment of specifically expressed genes identifies disease-relevant tissues and cell types. *Nat Genet*. 2018;50(4):621-629. doi:10.1038/s41588-018-0081-4

2. Bulik-Sullivan B, Loh P-R, Finucane HK, et al. LD Score regression distinguishes confounding from polygenicity in genome-wide association studies. *Nat Genet*. 2015;47(3):291-295. doi:10.1038/ng.3211

3. Habib N, Avraham-Davidi I, Basu A, et al. Massively parallel single-nucleus RNA-seq with DroNc-seq. *Nat Methods*. 2017;14(10):955-958. doi:10.1038/nmeth.4407

4. Skene NG, Bryois J, Bakken TE, et al. Genetic identification of brain cell types underlying schizophrenia. *Nat Genet*. 2018;50(6):825-833. doi:10.1038/s41588-018-0129-5

5. Tume CE, Chick SL, Holmans PA, et al. Genetic implication of specific glutamatergic neurons of the prefrontal cortex in the pathophysiology of schizophrenia. *Biological Psychiatry Global Open Science*. 2024;4(5):100345. doi:10.1016/j.bpsgos.2024.100345

6. Herring CA, Simmons RK, Freytag S, et al. Human prefrontal cortex gene regulatory dynamics from gestation to adulthood at single-cell resolution. *Cell*. 2022;185(23):4428-4447.e28. doi:10.1016/j.cell.2022.09.039

7. Cameron D, Mi D, Vinh N-N, et al. Single-Nuclei RNA Sequencing of 5 Regions of the Human Prenatal Brain Implicates Developing Neuron Populations in Genetic Risk for Schizophrenia. *Biol Psychiatry*. 2023;93(2):157-166. doi:10.1016/j.biopsych.2022.06.033

8. Yao S, Harder A, Darki F, et al. Connecting genomic results for psychiatric disorders to human brain cell types and regions reveals convergence with functional connectivity. *Nat Commun*. 2025;16(1):395. doi:10.1038/s41467-024-55611-1

9. Siletti K, Hodge R, Mossi Albiach A, et al. Transcriptomic diversity of cell types across the adult human brain. *Science*. 2023;382(6667):eadd7046. doi:10.1126/science.add7046
